# Evaluation of biochemical alterations produced by acetaminophen overdose in *Bubalus bubalis*

**DOI:** 10.14202/vetworld.2015.497-501

**Published:** 2015-04-16

**Authors:** Prashant Sudamrao Daundkar, Suresh Kumar Sharma

**Affiliations:** Department of Veterinary Pharmacology and Toxicology, College of Veterinary Science, Guru Angad Dev Veterinary and Animal Sciences University, Ludhiana, Punjab, India

**Keywords:** acetaminophen, biochemical, buffalo calves, liver dysfunction, renal impairment

## Abstract

**Aim:**

Evaluation of the effect of acetaminophen (APAP) overdose on biochemical parameters in buffalo calves.

**Materials and Methods:**

The experiment was conducted on six healthy male buffalo calves of 6-12 months age. The APAP was administered intramuscularly at the dose rate of 250 mg/kg body weight (B.W.) on day 0, followed by two subsequent doses at the dose rate of 50 mg/kg B.W. on day 2 and 4, respectively. Biochemical parameters including alanine aminotransferase (ALT), alkaline phosphatase (ALP), amylase, blood urea nitrogen (BUN), creatinine, and total acid phosphate were estimated in the plasma samples collected on 0, 1, 2, 3, 4, 5, and 6th day from the start of treatment.

**Results:**

Significant increase in the plasma levels of ALT (446.0%), ALP (137%), BUN (216.8%) and creatinine (149.2%) was recorded on day 3, 4, 3, and 4, respectively, after the start of APAP dosing. However, a significant decrease was observed in amylase activity with a maximum decline of 48.3% on 6th day after the start of treatment. No significant alteration was observed in ACP activity after APAP overdose.

**Conclusion:**

Administration of APAP in overdose produced hepatic dysfunction as evidenced by a significant increase in the activities of ALT and ALP, whereas reduced amylase may indicate acute pancreatitis in buffalo calves. In addition, repeated dosing also resulted in renal impairment in these animals as seen by a significant elevation in BUN and creatinine levels, whereas negligible effect on prostatic function.

## Introduction

Acetaminophen (APAP) is a regularly used non-narcotic analgesic as well as antipyretic agent in veterinary practice [1]. It has been reported that these effects of APAP are due to selective inhibition of cyclooxygenase (COX)-III, a variant of COX-I in the central nervous system and consequential inhibition of prostaglandin synthesis [2]. It is a safe drug when administered at therapeutic doses, but its overdose is quite common as it has narrow therapeutic index. Its overdose can lead to hepatic and renal damage in both humans and experimental animals [3].

The liver is a vital organ that regulate metabolism of xenobiotics including antimicrobials and also responsible for maintenance of body homeostasis. A number of chemical agents and drugs which are used routinely for the treatment including APAP produce cellular and metabolic liver damage [4,5].

APAP gets metabolized by conjugation with sulfate and glucuronidate and are excreted in the urine as an inert conjugate. Depending on the dose, a fraction of APAP is converted into N-acetyl-p-benzoquinone imine (NAPQI) - a highly reactive toxic intermediate by several P450 cytochromes. Significant amounts of NAPQI are efficiently eliminated by conjugation with glutathione (GSH). However, after a large dose of APAP, there is the saturation of sulfonation reaction and subsequently accumulated NAPQI depletes GSH in the liver. Unconjugated NAPQI binds to proteins and cellular structures and produces rapid cell death and necrosis that can lead to liver failure [5].

Kidney, another crucial organ for metabolism and excretion of many xenobiotics including antibiotics is at risk of APAP overdosing [3,6,7]. It is the second most sensitive organ of APAP toxicity and renal dysfunction occurs among patients with marked hepatic injury; though, nephrotoxicity after an acute overdose may even occur in the absence of hepatotoxicity [3].

Enzymes are proteins that catalyze the biochemical reactions in a sequence-specific manner. Exposure to APAP at high doses can cause cellular damage releasing these enzymes to plasma [3,4]. Thus, plasma enzyme levels can be used as a reliable marker for early diagnosis and prognosis of organ damage.

The use of NSAIDs is quite common in the treatment of bovines and its extra-label use may pose the risk of overdose in these species. Buffalo is an important dairy animal and contributes about 70% to the total milk production in India. Although effects of APAP overdose were evaluated in laboratory animals especially in rats, there were no reports to our perusal regarding the effect on liver as well as kidney in buffalo calves. The present study was therefore carried with the primary objective to see the effect of APAP overdose on biochemical parameters in buffalo calves.

## Materials and Methods

### Ethical approval

The study had been approved by the Institutional Animal Ethics Committee and experimental plan followed the ethical guidelines on the proper care as well as use of animals.

### Animals and treatment

The experiment was conducted on six healthy male buffalo calves of 6-12 months age and weighing between 100-130 kg. Animals were kept under normal ambient conditions in the experimental animal shed of the department and maintained on green fodder, concentrates, and wheat straw. Water was provided *ad libitum*. The dosage schedule of APAP (Paracetol-Vet, Cadila Health Care, India) was as per followed by Sharma and Ul Haque [8], i.e. 250 mg/kg body weight (B.W.) intramuscularly on day 0, followed by 2 subsequent doses at the dose rate of 50 mg/kg B.W. on day 2 and 4, respectively.

### Estimation of biochemical parameters

To study the biochemical parameters, blood samples were collected in heparinized vials from the jugular vein of animals on 0, 1, 2, 3, 4, 5, and 6th day from the start of APAP dosing. Plasma was separated by centrifugation at 3000 rpm for 15 min. Biochemical parameters including alanine aminotransferase (ALT), alkaline phosphatase (ALP), amylase, blood urea nitrogen (BUN), creatinine were estimated using Bayer Autopack kits (Bayer Diagnostics India Ltd., Baroda, Gujarat), and total acid phosphate (TACP) was estimated using Accurex (Accurex Biomedical Pvt. Ltd. Mumbai).

### Statistical analysis

All these results were subjected to one-way Analysis of Variance and the significance was tested using Dunnett’s test. The significance was assayed at 5% (p<0.05) as well as 1% (p<0.01) levels [9]. These statistical calculations were carried out with SPSS 16.0 software.

## Results and Discussion

Therapeutic dose of paracetamol administered in buffalo calves was reported to be 50 mg/kg B.W. [10]. The dose of the APAP administered to the animals in the present investigation was toxic dose as confirmed by previous hepatic dysfunction model used by the authors to conduct pharmacokinetic studies in buffalo calves [8].

**Figure-1:**
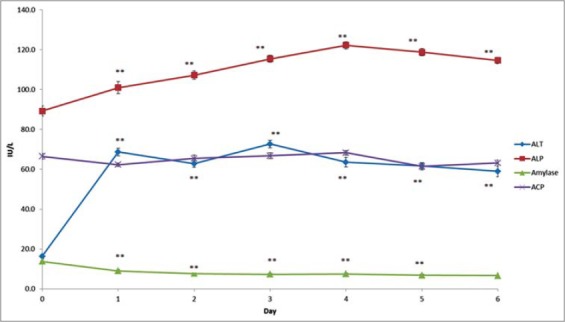
Effect of repeated administration of acetaminophen on plasma activity of alanine aminotransferase, alkaline phosphatase, amylase, and total acid phosphatase in buffalo calves, **mean significant difference (p<0.01 compared to control [0 day value]).

**Figure-2:**
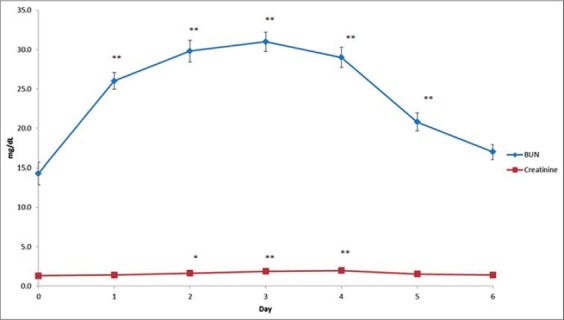
Effect of repeated administration of acetaminophen on plasma activity of blood urea nitrogen and creatinine in buffalo calves, *mean significant difference (p<0.05) and **means significant difference (p<0.01 compared to control [0 day value]).

Results on the effect produced by APAP overdosing on ALT, ALP, amylase, BUN, creatinine, and TACP are presented in Table-1 and also depicted in Figures-1 and -2, respectively. The values of these biochemical parameters on day 0 were taken as 100 and the increase/decrease calculated in % given in the parenthesis. Significant increase in the plasma ALT level was recorded with maximum of 446.0% on day 3, while amylase activity was significantly decreased from day 1 onward with maximum decline of 48.3% on day 6 after repeated APAP overdosing. The activity of ALP was elevated significantly from day 1 onwards with a maximum elevation (137%) on 4th day. In addition, significant elevation in BUN levels was observed from day 1 onwards with a maximum increase of 216.8% on day 3 following repeated administration of APAP. Similarly, creatinine levels also exhibited significant elevation from day 2 onwards with a maximum increase of 149.2% on day 4 after the start of treatment. However, no significant alteration was recorded in plasma TACP levels.

**Table-1:** Effect of repeated administration of APAP on plasma activity of ALT, amylase, BUN, creatinine, and TAD in buffalo calves.

Time after paracetamol administration (days)	ALT (IU/L)	ALP (IU/L)	Amylase (IU/L)	BUN (mg/dl)	Creatinine (mg/dl)	TAD (IU/L)
0	16.3±0.95 (100)	89.3±2.54 (100)	13.8±1.30 (100)	14.3±1.43 (100)	1.32±0.07 (100)	66.5±1.38 (100)
1	68.7±1.94** (421.5)	101.0±3.01** (113.1)	9.00±0.68** (65.2)	26.0±1.06** (181.8)	1.42±0.11 (107.6)	62.3±0.89 (93.7)
2	62.8±1.68** (385.3)	107.3±2.11** (120.2)	7.67±0.49** (55.6)	29.8±1.38** (208.4)	1.63±0.08* (123.5)	65.5±1.54 (98.5)
3	72.7±1.93** (446.0)	115.5±1.77** (129.3)	7.33±0.42** (53.1)	31.0±1.24** (216.8)	1.88±0.08** (142.4)	66.8±1.45 (100.5)
4	63.6±2.36** (390.2)	122.3±1.84** (137.0)	7.50±0.43** (54.3)	29.0±1.29** (202.8)	1.97±0.07** (149.2)	68.3±1.20 (102.7)
5	61.7±1.48** (378.5)	118.8±1.78** (133.0)	6.83±0.48** (49.5)	20.8±1.14** (145.5)	1.52±0.08 (115.2)	61.5±1.75 (92.5)
6	59.0±2.67** (362.0)	114.7±1.71** (128.4)	6.67±0.33** (48.3)	17.0±0.97 (118.9)	1.42±0.06 (107.6)	63.2±1.47 (95.0)

The values given are mean±SE of results obtained from 6 animals, Values in parentheses indicate the percentage of corresponding increase/decrease (0 day values taken as 100%),

* Means significant difference (p<0.05) and

** means significant difference (p<0.01 compared to control [0 day value]), SE=Standard error, APAP=Acetaminophen, ALT=Alanine aminotransferase, TAD=Total acid phosphatase, ALP=Alkaline phosphatase

Liver plays an important role in the biotransformation of xenobiotics by which toxic metabolites transformed into less harmful products and get excreted so as to reduce toxicity. However, during overdose these intermediate metabolites are produced in a large quantity resulting in liver cell damage and produce toxicity to this organ.

Hepatotoxicity is the most noteworthy feature of APAP overdose and it resembles other types of acute inflammatory liver diseases with a significant increase in levels of ALT. [11]. Elevated levels of ALT in the plasma often indicate the existence of medical problems for instance hepatitis, liver damage, bile duct problems, congestive heart failure, infectious mononucleosis or myopathy and diabetes [8]. The serum ALT is one of the most sensitive biochemical markers for the diagnosis of hepatic dysfunction [12]. During liver damage the transport function of hepatocytes is disturbed, resulting in consequential leakage of hepatic enzymes through plasma membrane therefore an elevation of aminotransferase activity in the extracellular fluid or plasma is considered as a sensitive indicator for hepatocellular damage [13]. The significant increase in the plasma ALT activity of APAP treated buffalo calves from day 1 to day 6 in the present study indicates liver impairment following its repeated administration [8,14].

ALP is a zinc-containing enzyme present in liver, biliary tracts, small intestines, bones, lungs, and kidney. Elevated levels in the blood may indicate damage to the liver and other organs [13,15]. The enhanced activity may be due to increased synthesis in response to hepatic damage as seen by the ALT levels in these buffalo calves after repeated administration of APAP [8].

Serum amylase is an important marker for acute pancreatitis and the reduced activity of this enzyme was reported after diazinon-induced acute pancreatitis in rats [16]. Similarly in the present study, the levels of amylase were reduced significantly in buffalo calves. The studies on pancreatectomized rats have already demonstrated the distribution of amylase in liver [17]. In addition, abnormal serum activity in patients with hepatic disease postulated that liver can be one of the sources of serum amylase [18]. Hepatic dysfunction as observed through increased ALT and ALP levels might be a secondary reason for decreased amylase activity in the present study. Thus, a significant decrease in the amylase activity in the present investigation might be because of reduced synthesis from damaged pancreatic and/or hepatic cells after repeated administration of APAP at high doses.

APAP gets eliminated mainly through the renal route. Thus, it is obligatory to examine the effects of its overdosing on kidney functions. Studies in the laboratory animals proposed that the overall incidence of acute renal failure after APAP poisoning is <2%, but can occur at the frequency of 10-40% in cases of severe hepatic necrosis. Renal prostaglandin production is mediated principally by cyclooxygenase and plays a pivotal role in compensatory renal hemodynamics. Severe adverse renal effects upon acetaminophen overdose might have occurred due to vasoconstriction subsequent to inhibition of renal prostaglandin-mediated vasodilatation, decreasing renal blood flow, and consequential reduction in glomerular filtration rate [19]. Hemodynamic depression following APAP administration occurs in a dose-dependent manner and it produces direct tubular toxicity through enhanced local production of the toxic quinoneimine intermediate metabolite and proximal convoluted tubular necrosis [6]. Some workers also proposed that APAP-induced nephrotoxicity due to covalent binding of reactive electrophiles [11].

BUN test is an estimate of the amount of nitrogen in the blood coming from urea, a waste product of the body. BUN levels increase in blood during renal insufficiency or high protein diet [13,20]. Blood urea nitrogen indicates the glomerular filtration but 75% of the kidney should be non-functional for BUN elevation [13]. Creatinine, a breakdown product of creatine phosphate in muscle, is an important indicator of renal health because it is an easily measured by product of muscle metabolism that is excreted unchanged by the kidneys [20]. Creatinine is considered to be an important biochemical parameter for the diagnosis of renal impairment [14]. Plasma creatinine levels help in evaluating renal functions particularly the glomeruli since creatinine is excreted in the urine after filtration. Increase in plasma BUN and creatinine levels in buffalo calves are suggestive of renal damage and may be attributed to urinary obstruction that potentiates decreased secretion of urea from the body after APAP overdose [6,7,13,14]. The BUN levels increased after the first dose with a maximum increase on day 3 followed by decreasing trend with non-significant increase on 6th day indicates kidneys compensatory mechanism to paracetamol overdose. This might be due to vasodilatation through cyclooxygenase mediated increased renal prostaglandin synthesis. Creatinine levels also followed the similar trend as that of BUN, although these were increased from day 2 onward up to day 4 and the percent increase was comparatively less than BUN.

It is well known truth that APAP overdose affect the hepatic functions but in present investigation authors evaluated its effect on second major organ of metabolism, i.e. kidney and observed renal impairment in addition to well-known hepatopathy in buffalo species as evident by alterations in the BUN and creatinine levels.

TAP, i.e. sum of both prostatic acid phosphatase (PAP) and non-PAP (NPAP) is a sensitive marker for the prostatic impairment especially prostatic adenocarcinoma in dogs [21]. The change in values of TACP in the plasma was non-significant as compared to day 0. This clearly indicates that APAP administration at high doses have a negligible effect on the prostatic function in buffalo calves.

## Conclusions

Repeated administration of APAP produced hepatic dysfunction as observed by a significant increase in the activities of ALT and ALP in the plasma of buffalo calves. Significantly reduced plasma amylase activity indicates the hepatic damage in addition to acute pancreatitis. Repeated high doses of APAP also resulted in renal impairment in these animals as seen by a significant elevation in the levels of BUN and creatinine, whereas negligible effect on prostatic function.

## Authors’ Contributions

PSD carried out the experiment, analyzed the samples, and drafted the final manuscript whereas SKS designed the study and reviewed the manuscript. Both authors read and approved the final manuscript.
